# From genetic improvement to commercial-scale mass culture of a Chilean strain of the green microalga *Haematococcus pluvialis* with enhanced productivity of the red ketocarotenoid astaxanthin

**DOI:** 10.1093/aobpla/plt026

**Published:** 2013-05-10

**Authors:** Patricia I. Gómez, Ingrid Inostroza, Mario Pizarro, Jorge Pérez

**Affiliations:** 1 FICOLAB, Grupo de Investigación Microalgal, Departamento de Botánica, Facultad de Ciencias Naturales y Oceanográficas, Universidad de Concepción, Chacabuco s/n, Barrio Universitario, Concepción, Chile; 2 Pigmentos Naturales S. A., Camino acceso a Pica s/n, Iquique, Chile

**Keywords:** Astaxanthin, commercial-sized open ponds, *Haematococcus pluvialis* mutant, North Chile, random mutagenesis.

## Abstract

Astaxanthin is a red ketocarotenoid, widely used as a natural red colourant in marine fish aquaculture and poultry and, recently, as an antioxidant supplement for humans and animals. The green microalga *Haematococcus pluvialis* is one of the richest natural sources of this pigment. However, its slow growth rate and complex life cycle make mass culture difficult for commercial purposes. The aims of this research were (i) to standardize and apply a genetic improvement programme to a Chilean strain of *H. pluvialis* in order to improve its carotenogenic capacity and (ii) to evaluate the performance of a selected mutant strain in commercial-sized (125 000 L) open ponds in the north of Chile. *Haematococcus pluvialis* strain 114 was mutated by ethyl methanesulfonate. The level of mutagen dose (exposure time and concentration) was one that induced at least 90 % mortality. Surviving colonies were screened for resistance to the carotenoid biosynthesis inhibitor diphenylamine (25 µM). Resistant mutants were grown in a 30-mL volume for 30 days, after which the total carotenoid content was determined by spectrophotometry. Tens of mutants with improved carotenogenic capacity compared with the wild-type strain were isolated by the application of these standardized protocols. Some mutants exhibited curious morphological features such as spontaneous release of astaxanthin and loss of flagella. One of the mutants was grown outdoors in commercial-sized open ponds of 125 000 L in the north of Chile. Grown under similar conditions, the mutant strain accumulated 30 % more astaxanthin than the wild-type strain on a per dry weight basis and 72 % more on a per culture volume basis. We show that random mutagenesis/selection is an effective strategy for genetically improving strains of *H. pluvialis* and that improved carotenogenic capacity is maintained when the volume of the cultures is scaled up to a commercial size.

## Abstract (precis)

There is a great breach between research made at universities and applications of these “academic results” to commercial purposes. This research is a successful example of this interaction. We show that random mutagenesis/selection is an effective strategy for genetically improving strains of the astaxanthin-producing microalga, *H. pluvialis* and that improved carotenogenic capacity attained is maintained when the volume of the cultures is scaled up to a commercial size. This research allowed to the company dispose of an improved strain accumulating 30% more astaxanthin that the wild type strain (per dry weight basis) and a 72% more (per culture volume basis).

## Introduction

Astaxanthin is a red ketocarotenoid, highly prized as a pigment for fish aquaculture and, more recently, for human consumption because of proven antioxidant, anti-ageing, anti-inflammatory and immune-stimulating properties (Christiansen and Torrissen 1997; Lorenz and Cysewski 2000; Jin *et al*. 2006; Zhang *et al*. 2009).

At present, the global demand for this pigment is satisfied mainly by synthetic astaxanthin produced by DSM in The Netherlands (http://www.dsm.com) and by BASF in France (http://www.basf.com). The estimated production cost of synthetic astaxanthin is approximately US$1000 kg^−1^, and the market price is approximately US$2000 kg^−1^ (Guerin *et al*. 2003; Zhang *et al*. 2009). Chile is the second largest producer of farmed salmon, an industry that consumes large amounts of astaxanthin, mostly of artificial origin (Valenzuela 2005). However, the need to compete in the demanding international markets has increased the national interest in replacing the synthetic pigment with a natural counterpart.

*Haematococcus pluvialis* is a unicellular green alga able to accumulate large amounts of astaxanthin (4 % dry weight) under stress conditions (Bubrick 1991; Krishna and Mohanty 1998; Boussiba 2000). The life cycle of *H. pluvialis* is complex and involves at least four types of cells. Under stress conditions, astaxanthin biosynthesis is accompanied by morphological changes of the motile vegetative (green) cells into non-motile cysts (red), which represent a resting stage with a heavy resistant cellulose cell wall (Boussiba 2000). Astaxanthin is accumulated in the cytoplasm of cyst cells, providing protection against photo-inhibition and oxidative stress (Yong and Lee 1991; Kobayashi *et al*. 1997).

Leading companies that cultivate *H. pluvialis* as a source of natural astaxanthin in the world are Cyanotech Inc. (http://www.cyanotech.com) and Mera Pharmaceuticals (http://www.merapharma.com) in Hawaii, Algatechnologies Ltd in Israel (http://www.algatech.com), and Biogenic Co. Ltd (http://www.bgenic.com) and Fuji Chemical Industry Co. Ltd (http://www.fujihealthscience.com) in Japan. In Chile, there are three companies that cultivate *H. pluvialis* commercially: Atacama Bio Natural (http://www.atacamabionatural.com), Pigmentos Naturales S.A. (http://www.pigmentosnaturales.cl) and Alimtec (http://www.alimtec.com).

Although *H. pluvialis* is one of the richest sources of astaxanthin, its massive culture for commercial purposes has been little exploited because of its slow growth rate and complex life cycle (Fujii *et al*. 2006). Considerable scope therefore exists for developing more productive strains. These may be obtained by simple selection or by genetic manipulation. Their creation would encourage the commercial culture of this microalga as a natural source of astaxanthin.

Several genes involved in the biosynthesis of astaxanthin have been cloned from *H. pluvialis* and partially characterized. These genes include δ-isopentyl diphosphate isomerase (*ipi*), phytoene synthase (*psy*), phytoene desaturase (*pds*), lycopene β-cyclase (*lyc*), β-carotenoid oxygenase (*crtO*) and β-carotenoid hydroxylase (*crtR-b*). The transcripts of these genes have shown a parallel overexpression with increased astaxanthin accumulation under oxidative stress, which demonstrates transcriptional control of these genes in the biosynthesis of astaxanthin in *H. pluvialis* (Lotan and Hirschberg 1995; Sun *et al*. 1998; Grunewald *et al*. 2000; Steinbrenner and Linden 2001, 2003). Li *et al*. (2010) evaluated the transcriptional expression of these genes in *H. pluvialis* versus irradiance increase (from 50 to 600 µmol m^−2^ s^−1^). The results of this study indicated that astaxanthin biosynthesis primarily depends on the transcriptional control of the gene encoding crtR-b and, to a lesser extent, on the genes encoding ipi, pds, psy and crtO (in this participation order). In spite of the availability of this valuable information and advances in genetic improvement of *H. pluvialis* by site-directed mutagenesis and transgenesis (Steinbrenner and Sandmann 2006; Kathiresan and Sarada 2009; Potvin and Zhang 2010), these strategies are rarely used by companies cultivating *H. pluvialis* and microalgae.

Random mutagenesis has been successfully applied in the past to improve the productivity of various microalgal species with biotechnological applications (López Alonso *et al.* 1996; Zhang and Lee 1997; Meireles *et al*. 2002; Chatuverdi *et al.* 2004; Chatuverdi and Fujita 2006; Yen Doan and Obbard 2012), including *H. pluvialis* (Tjahjono *et al*. 1994; Chumpolkulwong *et al*. 1997; Tripathi *et al*. 2001; Chen *et al*. 2003). The main advantage of this approach is its technical simplicity with no need for information on the genes involved or their regulation. This experimental strategy includes a first stage where random mutants are generated and a second phase where mutants are selected under selection pressures imposed by chemical inhibitors of critical steps in the biosynthesis of the target metabolite.

The aims of the present research were (i) to standardize and apply a genetic improvement programme to a Chilean strain *H. pluvialis* in order to improve its carotenogenic capacity and (ii) to evaluate the performance of a selected mutant strain in large commercial-sized open ponds in northern Chile.

## Methods

### Strain origin and maintenance conditions

*Haematococcus pluvialis* (strain 114) was obtained from Pigmentos Naturales S.A.

The stock culture was maintained in Bristol medium (Starr and Zeikus 1987) under controlled laboratory conditions at a photon flux density of 40 µmol m^−2^ s^−1^ (photosynthetically active radiation), a photoperiod of 16:8 h (light:dark) and a temperature of 20 ± 2 °C.

### Mutagenesis

Cells of *H. pluvialis* taken from the logarithmic growth phase (∼30 000 cells mL^−1^) were exposed to ethyl methanesulfonate (EMS). The mutagen dose (concentration and exposure time) was standardized to one that induced >90 % mortality.

Ten millilitres of culture were treated separately with different concentrations of EMS solution (from 0.4 to 1 % w/v) for 60, 90 and 120 min. After the incubation, 1 mL of treated culture was centrifuged at 4000 rpm for 3 min, the supernatant was discarded and the cell pellet was re-suspended in 1 mL of sodium thiosulfate (0.16 M). Washing with sodium thiosulfate was repeated twice, and the cell pellet was re-suspended in 250 µL of Bristol medium and kept in a culture chamber for 24 h. Then, serial dilutions of each culture were prepared and plated on solid Bristol medium (made by supplementation of Bristol medium with agar-agar 0.75 %). Plates were maintained under controlled laboratory conditions. When colonies were visible (after 20–25 days), they were counted and the mortality percentage was calculated for each EMS concentration and exposure time in comparison with non-EMS-exposed cultures (0 % mortality).

### Screening of inhibitor-resistant mutants

The carotenoid biosynthesis inhibitor (and herbicide) diphenylamine was used to screen for mutants with potentially improved carotenogenic capacity. Each mutant colony was inoculated onto two sister plates: one containing just Bristol medium (control) and the other containing diphenylamine (25 µM). Each colony was transferred to each plate by picking it with a toothpick and inoculating it onto an identifiable position of the plate (a gridded template was used). When colonies on sister plates were visible (after 15 days), the growth of each colony on both plates was compared and colonies robustly growing on the inhibitor plate were isolated and re-checked for inhibitor resistance. Herbicide-resistant mutants, confirmed by re-checking, were selected and used to inoculate liquid Bristol medium for growth and carotenogenesis analysis.

### Culture conditions at laboratory level

Each strain (wild type and mutants) was grown in 50-mL flasks with 30 mL of optimal *Haematococcus* medium (Fabregas *et al*. 2000). Cultures were initiated with 15 000 cells mL^−1^ and maintained at 70 µmol photons m^−2^ s^−1^ and 21 ± 3 °C, without aeration but manually shaken twice a day. The biomass of each culture was harvested after 30 days of culture.

These experiments were carried out in the facilities of Pigmentos Naturales S.A. located in Pica, north of Chile.

### Outdoor culture in open ponds

Selected mutant strains from the laboratory trials were grown in small experimental open-raceway ponds with paddle wheels to circulate the culture. Cultures of 80 L volume (10cm depth) were established for each strain using a culture medium commonly used in the productive process of Pigmentos Naturales S.A. (Patent 43154, 2007). Cultures were initiated with 15 000 cells mL^−1^. The experiments were performed in summer (January), maximum and minimum temperatures were 29 and 15 °C, respectively, while the maximum irradiance recorded was 2500 µmol m^−2^ s^−1^.

Trials in 125 000-L open ponds (commercial size) were carried out using the wild-type strain and the most productive mutant. Gradual scaling up of these cultures included the following phases: cultures in 200-mL volume were the inoculum for 20-L flasks and these were used to inoculate sterile plastic bags of 1100 L. Two of these bags were used to inoculate an intermediate-sized open pond of 25 000 L. All these steps were carried out in a greenhouse (indoors, clean conditions).

Intermediate-sized raceways of 25 000 L were used to inoculate the outdoor 125 000-L open ponds. The cultures were maintained at 20cm depth for 8 days by continuous refilling of the evaporated water, then no more water was added in order to facilitate cyst formation and subsequent separation of the biomass by decanting. These experiments were carried out in March; the average temperature was 24 °C and the maximum irradiance was 2500 µmol m^−2^ s^−1^. Biomass was harvested after 15 days of growth.

To harvest the biomass some of the supernatant was removed in the raceway, and the cyst pulp was transferred to a settling tank where more supernatant was removed. The pulp was concentrated further by centrifugation at 2800 rpm in a basket centrifuge and then ground in the aqueous phase using a ‘ball mill’. Finally, a mix of antioxidants (formulated by Pigmentos Naturales S.A.) was added to the ground biomass and it was spray dried at 160 °C. These broken and dried cysts were subsequently analysed for astaxanthin content by high-performance liquid chromatography (HPLC).

These experiments were carried out in the facilities of Pigmentos Naturales S.A. located in Pica, north of Chile.

### Growth analyses

The growth of cultures was monitored by cell counting in a Neubaüer chamber and by dry weight determination.

For dry weight determination, 10 mL of cell cultures were filtered through pre-dried and weighed Millipore filters (0.45 µm), washed with distilled water and dried at 80 °C to constant weight. The biomass was obtained by subtracting the weight of the filters from the weight of filters with cells.

### Total carotenoid analysis

Five millilitres of culture were centrifuged at 4000 rpm for 5 min, the supernatant was removed and 2.5 mL of dimethyl sulfoxide were added to the cell pellet. Samples were suspended by vortex shaking, incubated at 50 °C for 15 min, re-vortexed and centrifuged at 4000 rpm for 5 min. The supernatant was transferred to a volumetric 25-mL flask, 5 mL of 90 % acetone were added, vortexed and centrifuged at 4000 rpm for 5 min, and the supernatant was retained in the same volumetric flask. The acetone extraction was repeated three more times. The volume of each pigment extraction was adjusted to 25 mL with 90 % acetone and the samples were diluted as required for total carotenoid estimation using spectrophotometry according to Wegmann and Metzner (1971).

### Astaxanthin analysis

Astaxanthin content in broken cysts was determined by HPLC. Twenty-five milligrams of dried *Haematococcus* algae powder were extracted with 10 mL of 90 % acetone, vortexed and centrifuged at 4000 rpm for 5 min. Extraction was repeated until the pellet became colourless. The volume of each sample was made up to 25 mL with 90 % acetone.

Then, 3 mL of extract were transferred to a test tube and 2 mL of 0.05 M Tris–HCl buffer were added and incubated at 37 °C for 2 min. Afterwards, 200 µL of the enzyme cholesterol esterase (Sigma C-9281, 10 000 units per g) at 3.4 units mL^−1^ in 0.05 M Tris–HCl pH 7.0 buffer were added. After incubating at 37 °C for 45 min with gentle mixing, 1 g of sodium sulfate decahydrate and 2 mL of petroleum ether were added and the mixture vortexed for 30 s and centrifuged for 3 min at 3500 rpm. The petroleum ether layer containing the carotenoid mixture was then removed to a test tube containing 1 g of anhydrous sodium sulfate. The petroleum ether carotenoid extract was decanted and dried under nitrogen gas and re-dissolved in 3 mL of mobile phase for HPLC analysis (82 : 18, hexane : acetone).

High-performance liquid chromatography was carried out using a Waters chromatograph fitted with a UV-Vis detector (model 2489) and a YMC30 reversed phase (ODS) column. The analysis was performed at room temperature with an isocratic solvent mixture of hexane : acetone (82 : 18 v/v) flowing at 1.2 mL min^−1^. Astaxanthin (Sigma Chemical Company #A 9335) was used as the standard.

## Results

One per cent (w/v) EMS and 90 min were the concentration and exposure time selected for mutant generation in this study. Lower EMS concentrations, in any exposure time, were unable to induce the required minimum mortality of 90 % considered necessary for survivor cells to be potentially mutated (Table 1). Colonies isolated under this treatment were selected for their ability to survive in diphenylamine (25 µM). The 13 mutants that survived these treatments were then isolated (Table 2).

**Table 1.** Concentration and exposure time to EMS and survival percentage of *H. pluvialis.*

EMS concentration (%)	Exposure time (min)	Survival percentage
0.6	60	51.4
0.6	90	44.2
0.6	120	44.2
0.7	60	22.1
0.7	90	26.4
0.7	120	50.8
0.8	60	28.5
0.8	90	34.8
0.8	120	24.1
0.9	60	42.8
0.9	90	31.2
0.9	120	15.1
1	60	33.9
1	90	10.7
1	120	0.8

**Table 2.** Growth parameters and total carotenoid content in the wild-type strain and various mutants grown at laboratory level.

Strain	Maximum cell density (cells mL^−1^)	Dry weight (g L^−1^)	Total carotenoids by dry biomass (%)
Wild-type strain	148 063	1.1	0.50
Mutant B12	143 771	0.6	0.75
Mutant B18	155 788	1.3	0.63
Mutant B19	137 333	1.1	0.63
Mutant B21	195 700	1.1	0.88
Mutant B23	213 296	1.8	0.63
Mutant B24	243 767	1.5	1.88
Mutant B28	229 175	0.9	1.12
Mutant B29	220 163	1.1	0.75
Mutant B30	105 146	1.2	0.75
Mutant Iqq4	124 888	0.6	0.50
Mutant Iqq6	130 038	1.4	0.75
Mutant Iqq7	136 046	1.1	0.50
Mutant Iqq13	90 983	0.5	0.25

**Figure 1.**
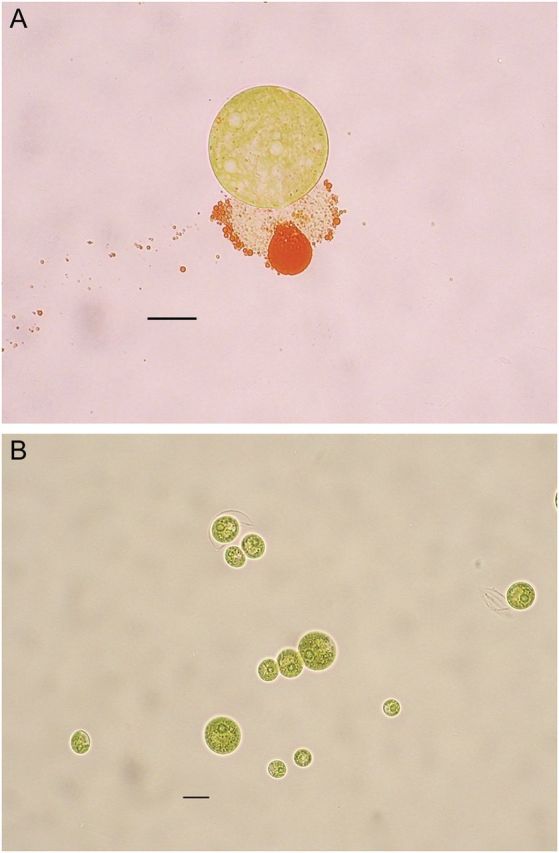
Morphological oddities detected among mutants isolated after mutagenesis with EMS. (A) Cyst of *H. pluvialis* ‘throwing out’ its carotenoids. (B) Non-motile vegetative green cells. Scale bars = 10 µm.

Some mutants exhibited curious morphological features such as spontaneous release of astaxanthin and loss of flagella (Fig. 1). The mutant able to ‘throw out’ its carotenoids (Fig. 1A) was not stable as its cells degenerated early (2 weeks) while the non-motile mutant (Fig. 1B) has remained stable for 2 years.

Table 2 shows the growth parameters and total carotenoid content in the wild-type strain and various mutants grown at laboratory level (30 mL).

Unlike the rest of the mutants, strains B12, B19, B24 and Iqq4 were easy to handle and maintained healthy cultures for at least 2 months, so they were selected to be grown in small experimental 80-L ponds. The growth parameters and total carotenoid content of these strains grown at this level are shown in Table 3.

**Table 3.** Growth parameters and total carotenoid content in the wild-type strain and four selected mutants grown in 80-L open ponds.

Strain	Maximum cell density (cells mL^−1^)	Dry weight (g L^−1^)	Total carotenoids by dry biomass (%)
Wild-type strain	42 500	0.4	0.75
Mutant B12	67 500	0.2	0.63
Mutant B19	37 500	0.1	1.5
Mutant B24	75 000	0.8	1.38
Mutant Iqq4	50 000	0.1	0.5

Mutant B24 was selected for its high biomass production and other desirable features, and when cultured in large 120 000-L open ponds this strain accumulated 30 % more astaxanthin than the wild-type strain (Table 4).

**Table 4.** Biomass (dry biomass by culture volume) and astaxanthin production of wild-type strain and mutant B24 grown in 120 000-L open ponds.

Strain	Dry weight (g L^−1^)	Astaxanthin by dry biomass (%)	Astaxanthin by culture volume (g m^3^)
Wild-type strain	0.28	2.03	5.68
Mutant B24	0.37	2.64	9.77

## Discussion

The global market for astaxanthin is worth more than US$200 million per year. About 130 tons of astaxanthin are consumed annually to feed the salmonids produced globally by aquaculture, of which >90 % is presently produced by chemical synthesis (Zhang *et al*. 2009; Li *et al*. 2011). However, despite chemical synthesis providing a stable source of synthetic astaxanthin, there is concern about its biological functions and food safety. Moreover, the high costs of synthetic astaxanthin and the growing market demand for natural astaxanthin to replace the synthetic pigment coupled with specific commercial applications (e.g. the nutraceuticals market) make mass production of this pigment from biological sources an attractive business opportunity. The latter becomes even more persuasive in the light of the high selling price of astaxanthin. Nutraceutical grade astaxanthin can cost US$100 000 kg^−1^ (Olaizola 2003).

Development of commercial cultures of *H. pluvialis* as an astaxanthin source requires highly productive strains. Considering the type of implementation and the professional profile of people working in microalgae production plants (e.g. factory workers, aquaculture engineers, chemical engineers), it is very unlikely that these companies will conduct studies on genetic improvement of strains independently. The present research has successfully addressed this shortcoming by means of collaboration between a university and a commercial enterprise.

The improvement in productivity of astaxanthin by *H. pluvialis* achieved through random mutagenesis–selection is promising (Table 4) when compared with previously reported outcomes. Chen *et al*. (2003) reported an increment from 1.2 to 2.5 % astaxanthin by dry weight (in their best mutant strain) when *H. pluvialis* was mutagenized using a strategy similar to ours. The increment of astaxanthin content achieved in the present study was from 2.03 to 2.64 % astaxanthin by dry weight; this corresponds to a 30 % increase on a per dry weight basis and a 72 % increase on a per culture volume basis (Table 4), highlighting that in our study the trials were conducted at a realistic commercial scale of culture.

In recent years, numerous studies on culture conditions and the selection of suitable strains for mass culture of *H. pluvialis* have been conducted (Kathiresan and Sarada 2009; Zhang *et al*. 2009; Li *et al*. 2011). Zhang *et al*. (2009) grew *H. pluvialis* in 20-m^3^ open-raceway ponds and achieved an astaxanthin content ranging from 1.61 to 2.8 % by dry weight, with an average astaxanthin content of 2.10 %. In our work, we grew wild-type and mutant strains in 120-m^3^ open-raceway ponds, a significantly higher culture volume, and obtained an astaxanthin production of 2 % for the wild-type strain and 2.6 % for the mutant strain B24 (Table 4). These are promising results considering that usually biomass microalgal productivity, and therefore productivity of any metabolite accumulated by the microalgae, decreases with the increase in culture volume.

In this work we demonstrated that random mutagenesis/selection is an effective strategy for genetically improving strains of *H. pluvialis*. Greater carotenogenic capacity achieved in small experimental cultures was maintained when cultures were scaled up to commercial size.

In spite of advances in genetic improvement of microalgae by means of genetic engineering procedures, mutants are more readily accepted than transgenics by consumers, since induced mutagenesis is a much more natural strategy that just speeds up a process also capable of occurring spontaneously.

## Conclusions

Genetic improvement of *H. pluvialis* by random mutagenesis–selection was demonstrated to be a successful strategy to increase the content of astaxanthin, a red ketocarotenoid commonly used as a natural red colourant in fish farming. Improved astaxanthin productivity of the mutant strain was maintained even when grown on a large scale and holds promise as the basis for viable commercial production of this valuable biochemical by natural means.

## Sources of Funding

This work was supported by the grant INNOVA Chile No. 206-5278: ‘Induction to mutagenesis in native strains of *Haematococcus pluvialis*, to increase its carotenogenic productivity at industrial scale’ given to Panades y Cia Ltda.

## Contributions by the Authors

P.I.G. was responsible for the data analysis and preparation of the manuscript. P.I.G., I.I., M.P. and J.P. contributed to setting up the experiments, performing the measurements and improving the manuscript.

## Conflicts of Interest Statement

None declared.
